# Integrative network analyses of wilt transcriptome in chickpea reveal genotype dependent regulatory hubs in immunity and susceptibility

**DOI:** 10.1038/s41598-018-19919-5

**Published:** 2018-04-25

**Authors:** Nasheeman Ashraf, Swaraj Basu, Kanika Narula, Sudip Ghosh, Rajul Tayal, Nagaraju Gangisetty, Sushmita Biswas, Pooja R. Aggarwal, Niranjan Chakraborty, Subhra Chakraborty

**Affiliations:** National Institute of Plant Genome Research, Aruna Asaf Ali Marg, New Delhi, 110067 India

## Abstract

Host specific resistance and non-host resistance are two plant immune responses to counter pathogen invasion. Gene network organizing principles leading to quantitative differences in resistant and susceptible host during host specific resistance are poorly understood. Vascular wilt caused by root pathogen *Fusarium* species is complex and governed by host specific resistance in crop plants, including chickpea. Here, we temporally profiled two contrasting chickpea genotypes in disease and immune state to better understand gene expression switches in host specific resistance. Integrative gene-regulatory network elucidated tangible insight into interaction coordinators leading to pathway determination governing distinct (disease or immune) phenotypes. Global network analysis identified five major hubs with 389 co-regulated genes. Functional enrichment revealed immunome containing three subnetworks involving CTI, PTI and ETI and wilt diseasome encompassing four subnetworks highlighting pathogen perception, penetration, colonization and disease establishment. These subnetworks likely represent key components that coordinate various biological processes favouring defence or disease. Furthermore, we identified core 76 disease/immunity related genes through subcellular analysis. Our regularized network with robust statistical assessment captured known and unexpected gene interaction, candidate novel regulators as future biomarkers and first time showed system-wide quantitative architecture corresponding to genotypic characteristics in wilt landscape.

## Introduction

Plants frequently encounters to wide range of patho-stresses that modulate growth and development thereby affecting the overall productivity. Counter action strategies are pre-requisite for assault and defense against virulence factors of pathogen and innate immune system of plants. Several of these stresses are united by the fact that at least part of their detrimental effect on plant performance is caused by the deregulation of the immune status. It is a monolayer paradigm in which mounting resistance in host is counter-balanced by deregulated pathogen virulence. Although sequence of recognition, signal transduction and response is common theme but selection in plant is unique to type of pathogen attack contributing to disease or resistance^1^. Plant innate immunity can be activated by microbe, chitin or pathogen-associated molecular patterns (M/C/PAMPs) in accurate manner and determined by transcription factors and extensive transcriptional reprogramming. Fungal invasion is orchestrated by set of genes that exhibit induction and/or repression during infection dictating host’s ability to mitigate and pathogen to propagate in plant-microbe interactions^2^. Thus, host immunome and diseasome should be precisely regulated by gene networks to maintain balance between immune response and pathogen colonization. Identification of genotype specific molecular events may provide unique insight into the effect of genotypic variation on the plant-pathogen interaction. However, the difference and the overlap in the downstream components of CTI, PTI and ETI are largely unknown^1,3–10^.

Chickpea (*Cicer arietinum*) is the second most important legume worldwide, but its production is highly threatened due to vascular wilt. *Fusarium oxysporum*, causal agent of vascular wilt is a soil borne fungus and adapt hemibiotrophic mode of invasion. According to host specificity, wilt-inducing *F*. *oxysporum* isolates are categorized into >120 *formae speciales* (f. spp.)^11^. Host genotype specificity and hemibiotrophic pathogen interaction depends on gene-for-gene model^12–14^. Despite earlier study showing that resistance to *Fusarium* in chickpea is host specific, polygenic and under complex genetic control^15^, the mechanism controlling effective resistance and genotype-pathotype interaction remains largely unknown.

Advances in high-throughput omics technologies offer unparalleled opportunities to evaluate patho-stress response at system level. These responses are characterized by dynamic and variable gene expression changes leading to reprogramming of many cellular functions^16^. It is known that differences in the transcriptome between PTI and ETI are largely quantitative^17,18^. Further, interconnectivity between subcellular compartments dictate organelle specific gene interaction spread along the gene regulatory network modules^19^. Various schemes have been proposed from differential transcriptome to identify features/genes that are dramatically different in disease and immune state^20^. Consequently, network-based protein-protein interaction (PPI) characterize intricate and interwoven relationships that govern cellular functions. Thus, integrating PPI and gene-expression profile provides novel insights into functional interactions amongst deregulated genes^21–25^. This combined approach can provide insights into regulation of cellular processes and identify the interaction architecture and the underlying gene regulatory networks^26^. To elucidate molecular mechanism of diseasome or immunome conventional statistical methods and computational approaches have been employed^27,28^. Regulatory relationships among 22 immune-related genes in *Arabidopsis* were elucidated based on sector switching model derived from network analysis^29^. Thus, transcript profiling assist in elucidating logic of regulatory circuits to provide insights into cellular processes and identification of interaction architecture to manage plant immune response^30^. Earlier, we reported expressed sequence tags (EST) and comprehensive insight into identity and function of immune responsive root transcriptome in chickpea^31^.

Here, we present integrative functional network analyses based on cDNA microarray temporal datasets consisting of 6072 spots representing 1749 unigenes to examine the common and discrete features of chickpea gene network during host specific resistance in response to *Fusarium* wilt. The aim was to create a signal transduction catalogue for chickpea defense and/or disease signaling and develop a snapshot of transcriptional regulatory programs underlying the immune response. We assembled gene network by integrating temporal gene expression data from two contrasting chickpea genotypes differing in patho-stress response. Data analysis revealed that molecules belonging to several biological processes were preferentially and differentially expressed during patho-stress. A diseasome and immunome was constructed to reveal invasion or resistance mechanism. Corollary of this hypothesis was elucidated in unbiased manner to determine significant difference in gene expression and interdependencies among cellular components to determine relationships among variables. Further, the host specific response seemed to be tightly regulated by transcriptional regulators to execute condition-specific and complex biological functions in eukaryotes. Finally, the study underpins genotype dependent transcriptional regulation during patho-stress and highlights the importance of module coordination in host specific plant defense.

## Results

### *Fusarium* subverts physiological processes in chickpea

**Figure 1:**
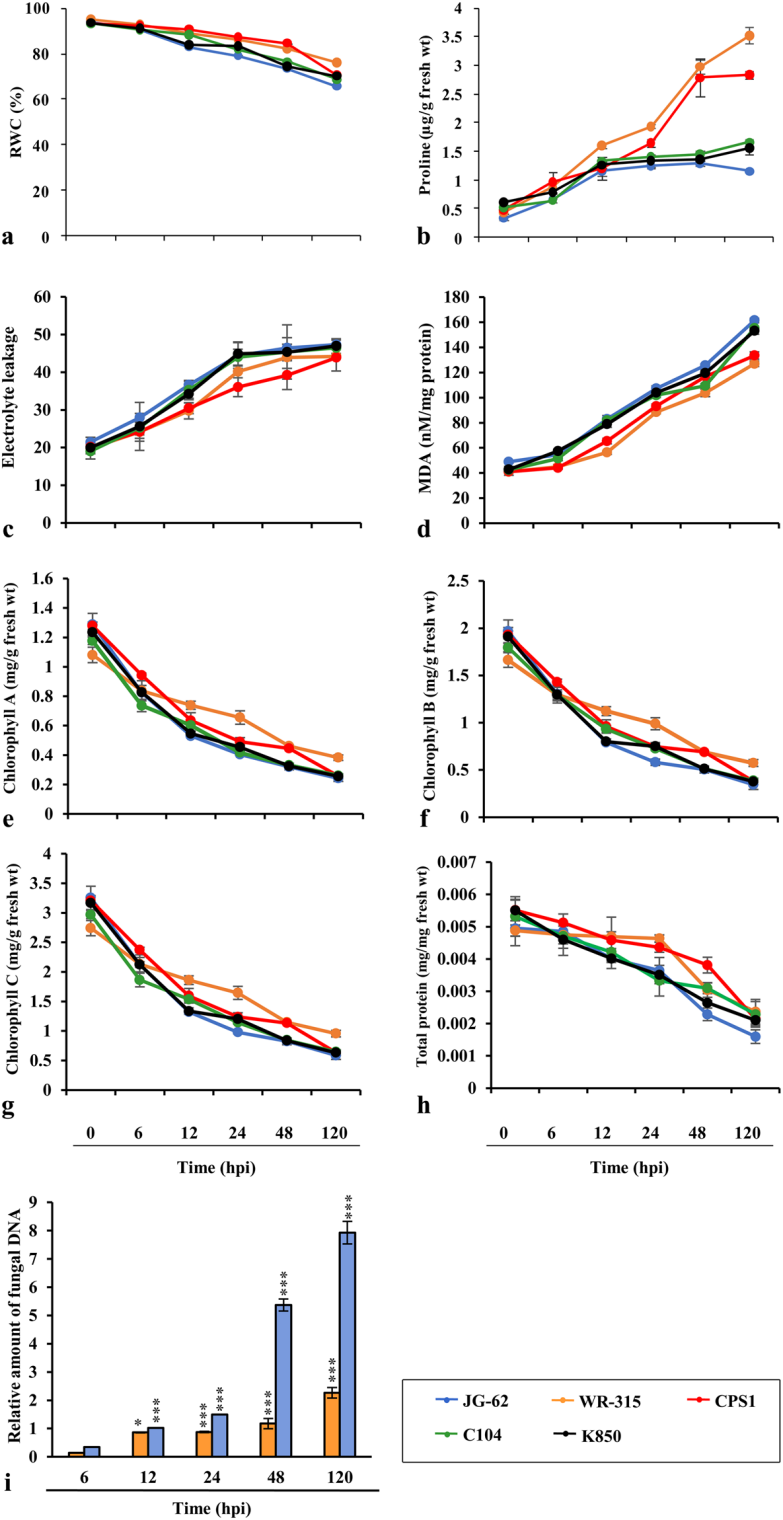
Physiological and biochemical analysis of chickpea varieties in response to *Fusarium* attack. (**a**) percent RWC, (**b**) endogenous free proline content, (**c**) estimation of electrolyte leakage, (**d**) MDA levels, (**e**,**f**,**g**) measurement of photosynthetic pigments chlorophyll A, chlorophyll B and chlorophyll C, respectively, (**h**) total protein, (**i**) relative quantification of fungal biomass by real-time PCR on DNA extracted from *F*. *oxysporum*-infected roots of JG-62 and WR-315 at 6, 12, 24, 48 and 120 hpi. Amplification values for FoGDP were normalized to the abundance of chickpea 18S sequence. Each replicate is a pool of five plants of three independent experiments with three biological replicates. Lines and vertical bars denotes the mean values ± SE. Expression changes were analyzed by ANOVA and Tukey post-hoc test (p < 0.05) and vertical bars indicate SE. “*” indicates statistical significance of relative amount of fungal DNA.

*Fusarium* infects through roots, enters xylem vessels, produce pathotoxins affecting plant metabolism. Furthermore, pathogen invasion also induce membrane injury and affect water conductance and potential that regulate stomatal opening and obstruct water and mineral transport leading to wilting and subsequent death of host plant^32,33^. To investigate genotype specific cellular responses during host specific resistance, we screened different chickpea varieties challenged with *Fusarium oxysporum*. Sharply contrasting phenotype and considerable symptomatic differences were observed amongst the studied genotypes. WR-315 and CPS1 appeared to be the most resistant variety while JG-62 was found to be most susceptible and early wilting genotype. C-104 and K850 showed late wilting phenotype. No symptoms were observed on mock-inoculated seedlings. No visible changes were observed in WR-315 seedlings till one month post inoculation, but in JG-62 wilting started after 48 hpi and the symptoms were further intensified at 96–120 hpi exhibiting severe wilting. To understand pathogen progression quantitatively, fungal biomass was determined in *Fusarium* challenged resistant and susceptible chickpea genotypes. We found significant increase in fungal biomass over time in susceptible genotype (JG-62) in contrast to the resistant genotype (WR-315) (Fig. 1i). Furthermore, we examined ROS production in both the genotypes till 120 hpi as qualitative measure. A large ROS burst was observed in patho-stressed resistant WR-315 roots at 12–48 hpi, while ROS production was less pronounced in the susceptible genotype, JG-62. *Fusarium* induced ROS production was not detected in control roots by DAB staining (Supplementary Fig. S1).

Plants adapt to water stress condition by increased concentration of intracellular solutes, such as proline, which facilitate the maintenance of cell pressure potential^34^. Moreover, role of proline as osmoprotectant in plant defense against invading pathogen has earlier been documented^35,36^. We observed that WR-315 was able to maintain Relative Water Content (RWC) considerably at higher level while JG-62 showed the maximum decline, suggesting inability of susceptible genotype to take up water due to maximum colonization of the pathogen in the xylem (Fig. 1a). Concomitantly, WR-315 showed significant increase in endogenous free proline followed by CPS1 during patho-stress. This may be related to ~90% recovery in RWC (Fig. 1b). Plasma membrane integrity and lipid peroxidation due to fungal attack often leads to pathogen invasion. Marked difference in electrolyte leakage was observed between genotypes with maximum in JG-62 and least in WR-315 and CPS1 (Fig. 1c). Lipid peroxidation also showed a similar trend with increased malondialdehyde (MDA) levels (~4.5-fold) in JG-62 (Fig. 1d). Photosynthetic ability is one of the most significant parameter in stress response^37^. WR-315 and CPS1 maintained significantly higher chlorophyll a, chlorophyll b and chlorophyll c in addition to protein content than other genotypes (Fig. 1e–h). Taken together, these results confirm WR-315 as most resistant and JG-62 as most susceptible to Fusarium wilt which has earlier been well documented^38–44^.

### Mutual exclusivity and consistency of transcriptional reprogrammers in disease vs immune state

**Figure 2:**
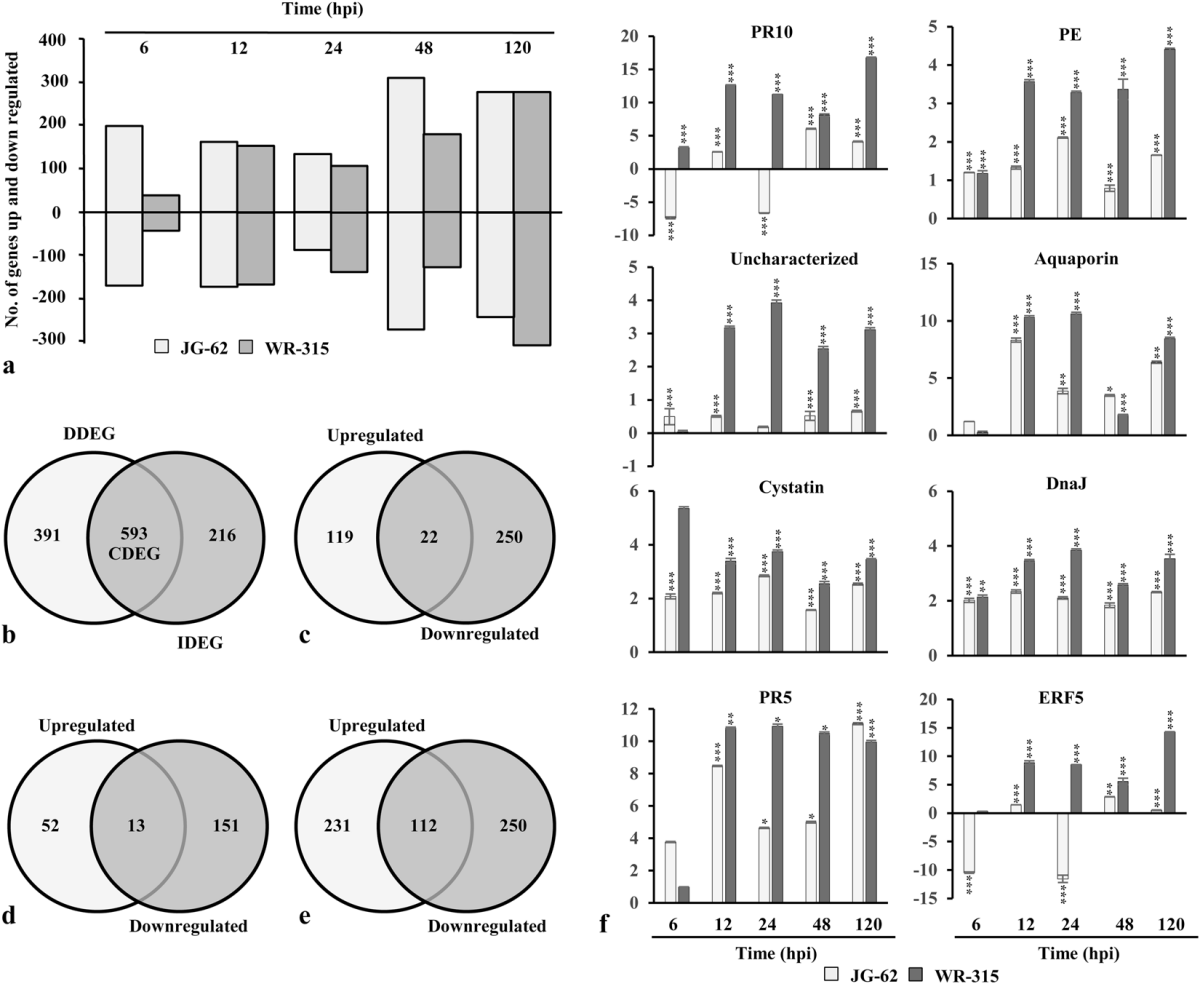
Gene expression pattern of DEGs and qRT-PCR analysis. (**a**) regulation of DEGs for JG-62 (wilt susceptible) and WR-315 (wilt resistant) genotypes of chickpea over the time course after inoculation with *Fusarium*, (**b**) venn diagram depicting exclusive and overlapping DEGs. (**c**,**d**,**e**) venn diagram representing regulation of IDEGs, DDEGs and CDEGs. (**f**) relative mRNA levels of eight candidate DEGs involving PR10, pectinesterase (PE), uncharacterized protein, aquaporin, cystatin, DnaJ, PR5, ERF5 were assessed by qRT-PCR. Statistical significance of expression changes were analyzed by ANOVA and indicated by * for p < 0.05 (Tukey post-hoc test). Vertical bars denote SE.

Temporal gene expression profiles in *Fusarium* infected chickpea seedlings of two contrasting genotypes, wilt-susceptible (JG-62) and wilt-resistant (WR-315) were assessed using microarray analysis. Among 6072 qualified probes representing 1749 genes present in the microarray, compared to the control mock inoculated, a set of 1200 immune responsive factors (IRFs) and 77 fungal genes were differed by a factor of two or more (fold change > 2.5, p < 0.05) in at least one of the six time points in either genotypes (Supplementary Dataset 1; Fig. 2a). From the identified 77 fungal genes, 17 were exclusively expressed in JG-62, including 11 downregulated and 6 upregulated genes. 11 fungal genes (4 downregulated and 7 upregulated) were specifically expressed in WR-315. However, 43 fungal genes with 12 and 31 genes exhibiting downregulation and upregulation, respectively were differentially expressed in both the genotype. Three fungal genes, namely exosome complex component, mtr3 and uncharacterized fungal genes exhibited upregulation in JG-62 but downregulated in WR-315. A fungal ATP synthase subunit showed downregulation at early time points and was upregulated in later time point in JG-62, whereas in WR-315 it showed upregulation till 120 hpi. Gene with unknown function and v-type proton ATPase catalytic subunit A were upregulated in early time point and downregulatd in later time point in JG-62, whereas in resistant genotype they showed upregulation till 120 hpi or showed no expression, respectively. Of the IRFs, 391 disease associated differentially expressed genes (DDEGs) and 216 immunity associated differentially expressed genes (IDEGs) were unique to JG-62 and WR-315, respectively while 593 common differentially expressed genes (CDEGs) were found to be shared between the genotypes (Fig. 2b). The analysis revealed that each stage of disease development in JG-62 or immune response in WR-315 was represented by distinct transcription profile. Out of 391 DDEGs in JG-62, 119 genes were upregulated and 250 were downregulated (Fig. 2c). In WR-315, among 216 IDEGs 52 exhibited upregulation and 151 showed downregulation (Fig. 2d). Of note, although in both genotypes, percentage of up- or down-regulated DEGs was different but the number was similar at each post-inoculation time. Meanwhile, the total up- and down-regulated DEGs were comparable between two genotypes. However, in JG-62, more number of genes (337 DEGs) showed altered expression at 6 hpi than WR-315 (75 DEGs) possibly due to the fact that pathogen sensing and perception promote gene expression regulation in susceptible genotype at early stage of invasion. Similar number of genes were differentially regulated at 12 hpi and 24 hpi in both the genotypes reflecting the transition point for disease or immune state. At 48 hpi, 537 DEGs were identified in JG-62 and 283 DEGs in WR-315, suggesting that in later stages clear signal separation represented by distinct transcriptional reprogrammers might regulate disease or immune pathways in contrasting genotypes. Interestingly, opposite trend in gene expression was observed at 24 hpi showing more upregulated DEGs and 120 hpi depicting more downregulated IDEGs in resistant genotype confirming a link between number of DEGs and immune processes. To further assess the similar or diverse effects of the two contrasting genotypes, venn diagrams were used to depict the overlap of CDEGs that were significantly up- and down-regulated. Among upregulated CDEGs, 231 genes and in the downregulated CDEGs, expression of 250 genes was commonly regulated in both genotypes (Fig. 2e). While 28 were preferentially upregulated in JG-62 and downregulated in WR-315 and 19 showed upregulation in WR-315 and downregulation in JG-62. Furthermore, in both the genotypes, cell wall remodelers like pectinesterase and xyloglucosyl transferase were upregulated while cytoskeleton associated genes like actin and profilin-like genes were downregulated. Also ROS associated genes like cytochrome P450, cytochrome P450 monooxygenase and peroxidase showed upregulation in both genotypes (Supplementary Dataset 1). This is consistent with the fact that distinct counter regulatory pathways affect the set-point in immune/disease homeostatic control that is governed by the net balance between inhibitory and stimulatory responses in two genotypes.

**Figure 3:**
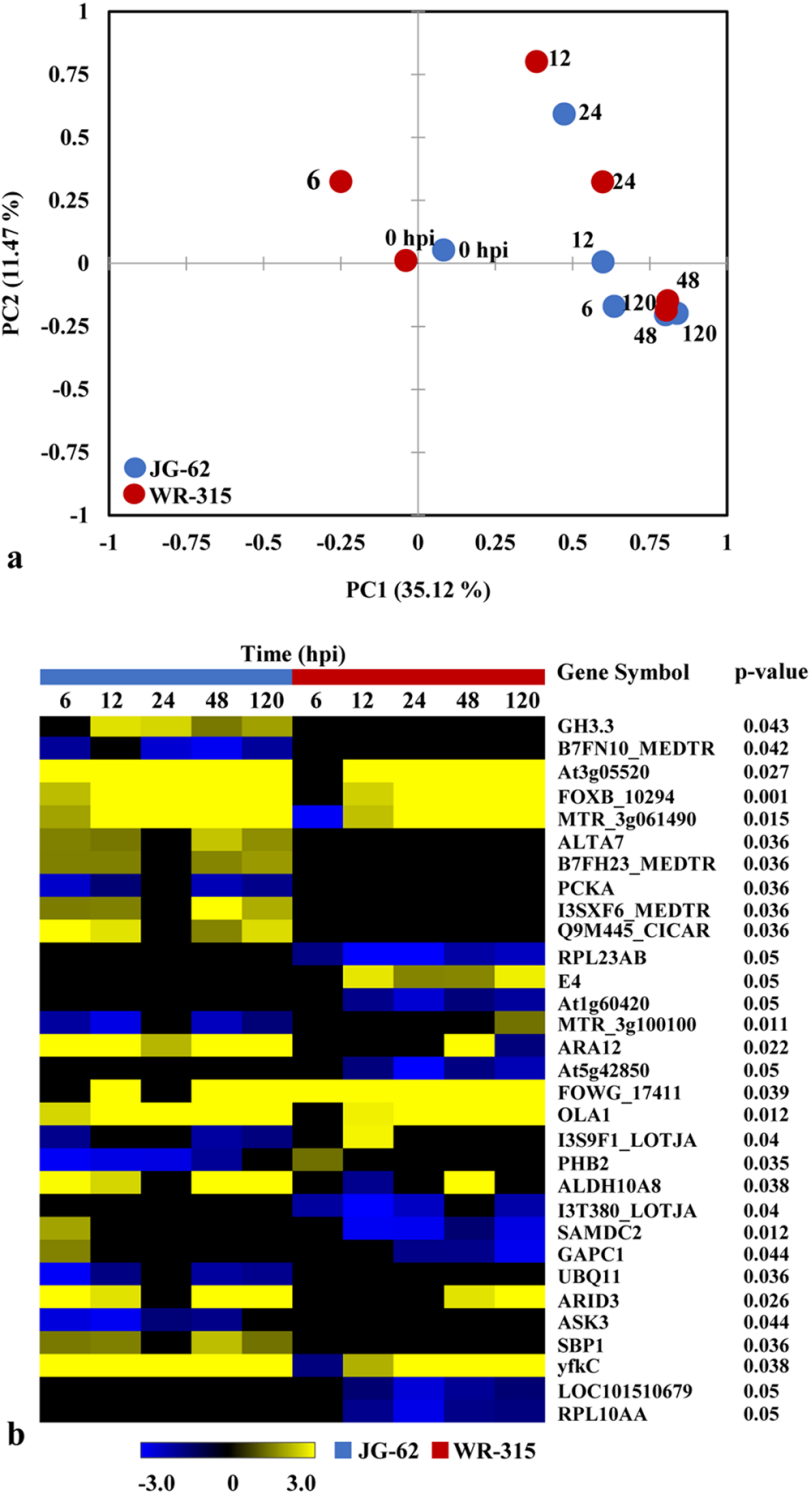
Investigation of identified DEGs. (**a**) PCA of the data set shows that the expression profiles of all 12 conditions are different from each other. X-axis and y-axis denotes principal component 1 (PC1) and principal component 2 (PC2), respectively. Number refers to IDs depicted in Supplementary Table S1. (**b**) Heat map of DEGs between two genotypes. Significant differences (p < 0.05) were estimated using two-way ANOVA.

Principal component analysis (PCA) signifies variation in gene expression in which each dimension represents the variability^45^. We conducted PCA to evaluate the degree of differences and relatedness of transcription profiles among different conditions. It was observed that maximum variation was accounted for by first component PC1 (eigenvalue 13.698; 35.12%) and second component PC2 (eigenvalue 2.428; 11.47%). Although 2-D plot revealed similar variance across time points in both early and later stages of invasion, but the transcripts were of variable nature between the genotypes (Fig. 3a). Further, to identify genes that significantly regulate expression during patho-stress, we conducted two-way ANOVA and found 31 genes that had significant expression difference between the genotypes (Fig. 3b).

Next, to facilitate biological interpretation of the dataset, we generated an unbiased framework that group similar expression across time points and genotypes using SOTA analysis (Supplementary Fig. S2). Clustering of DDEGs, IDEGs and CDEGs resulted in ten distinct clusters distinguishing the five JG-62-treated and five WR-315-treated seedlings and the two remaining untreated seedlings from each of the control genotype. Lists of commonly and differentially expressed genes are provided in Supplementary Datasets 2, 3, 4. According to the gene expression pattern of two contrasting genotypes, we classified the DEGs into two subsets of SOTA clusters (SC): (i) DDEGs exclusive to JG-62 representing SC1, SC3, SC5 and SC8 and (ii) IDEGs exclusive to WR-315 representing SC4 and SC7. Further analysis of the CDEGs identified 266 genes that act as core genes shared by JG-62 and WR-315 confirming that combinatorial interactions of DEGs drive reprogramming events during patho-stress.

### Validation of microarray data as recurrent transcript

The effect of genotype and temporal kinetics under patho-stress was further assessed by qRT-PCR (Fig. 2f, Supplementary Table S1). IDEGs like PR10, pectinesterase, uncharacterized protein showed elevated expression in WR-315. Aquaporin, a DDEG was markedly repressed in JG-62. Transcripts belonging to CDEGs, namely cystatin and DnaJ exhibited upregulation or mixed expression while PR4 showed repression in both genotypes. Notably, ERF5 involved in hormone signaling showed upregulation in susceptible genotype and downregulation in resistant genotype. Thus, relative expression levels of DEGs showed strong positive correlation and similar trends as compared with microarray analysis (Supplementary Fig. S3).

### Cannonical and non-cannonical pathways modulated by patho-stress

**Figure 4:**
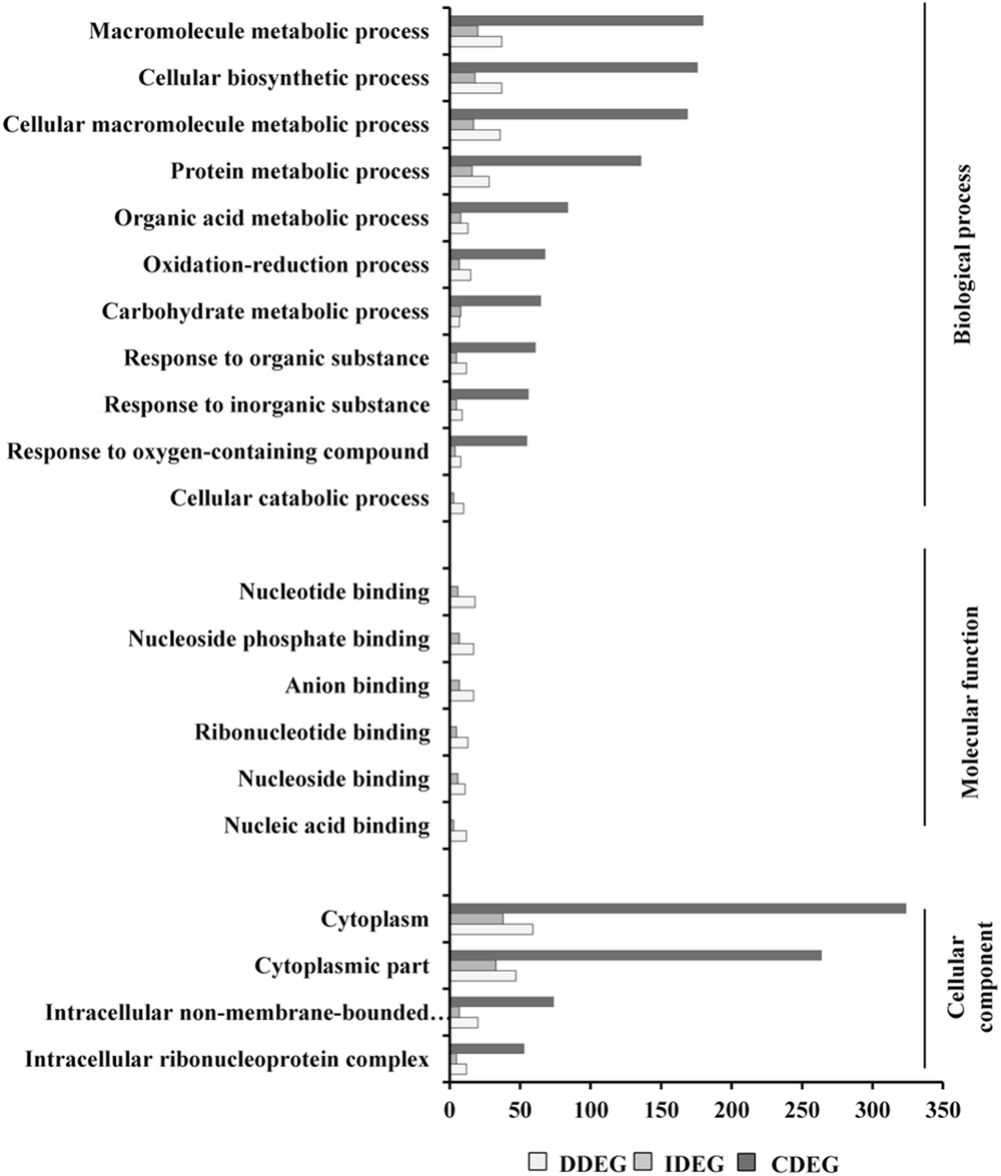
Functional enrichment of the DEGs. Distribution of transcripts based on Blast2GO analyses. Y-axis indicates significant Blast2GO functional categories (p < 0.05) and the X-axis shows number of transcripts.

To assess the biological relevance of the transcriptional reprogrammers, we performed gene ontology analysis of DEGs using Blast2GO^46^. Functional analysis of the DDEGs, IDEGs and CDEGs enriched GO terms belonged to biological process, cellular component and molecular function (Fig. 4). The DEGs in the cluster of biological process was primarily involved in cellular metabolism (251 IDEGs, 432 DDEGs, 2329 CDEGs) and oxidation reduction process (7 IDEGs, 15 DDEGs, 74 CDEGs). This is consistent with the fact that carbon and nitrogen metabolism were greatly affected during patho-stress irrespective of genotypes^47,48^. The DEGs were also found to be members of variety of cellular components with a large representation from cytoplasm. As JG-62 and WR-315 displayed distinct molecular properties in response to *Fusarium*, we intended to identify key genes involved in maintaining cellular functionality. It was observed that genes related to nucleic acid binding (3 IDEGs, 12 DDEGs, 56 CDEGs), nucleoside binding (6 IDEGs, 11 DDEGs, 78 CDEGs), ribonucleotide binding (5 IDEGs, 13 DDEGs, 81 CDEGs), anion binding (7 IDEGs, 17 DDEGs, 97 CDEGs) and nucleoside phosphate binding (7 IDEGs, 17 DDEGs, 102 CDEGs) may determine disease or immune state of the respective genotypes.

### Identification of gene network and mapping functional transcription factor networks during vascular wilt

**Figure 5:**
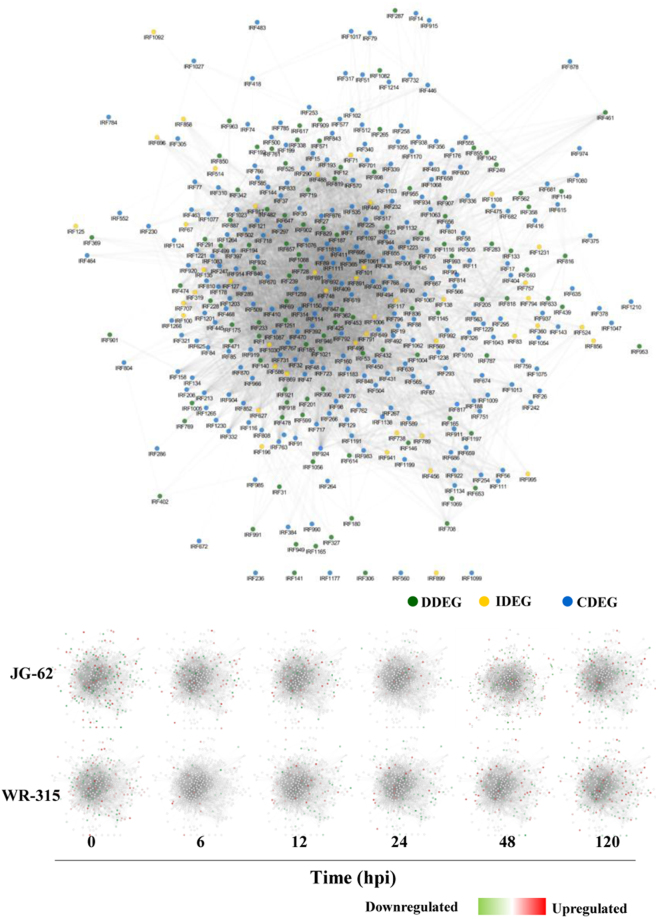
Gene network analysis. Network was constructed using gene expression data of 12 conditions from susceptible and resistant genotypes. Changes in expression profiles at different time points from two genotypes were captured in co-expression network. Each node represents a given protein associated with an EST (based on top BLAST hit against SwissProt) and an edge denotes a probability of two given proteins (nodes) potentially interacting based on the cytoprophet algorithm.

**Figure 6:**
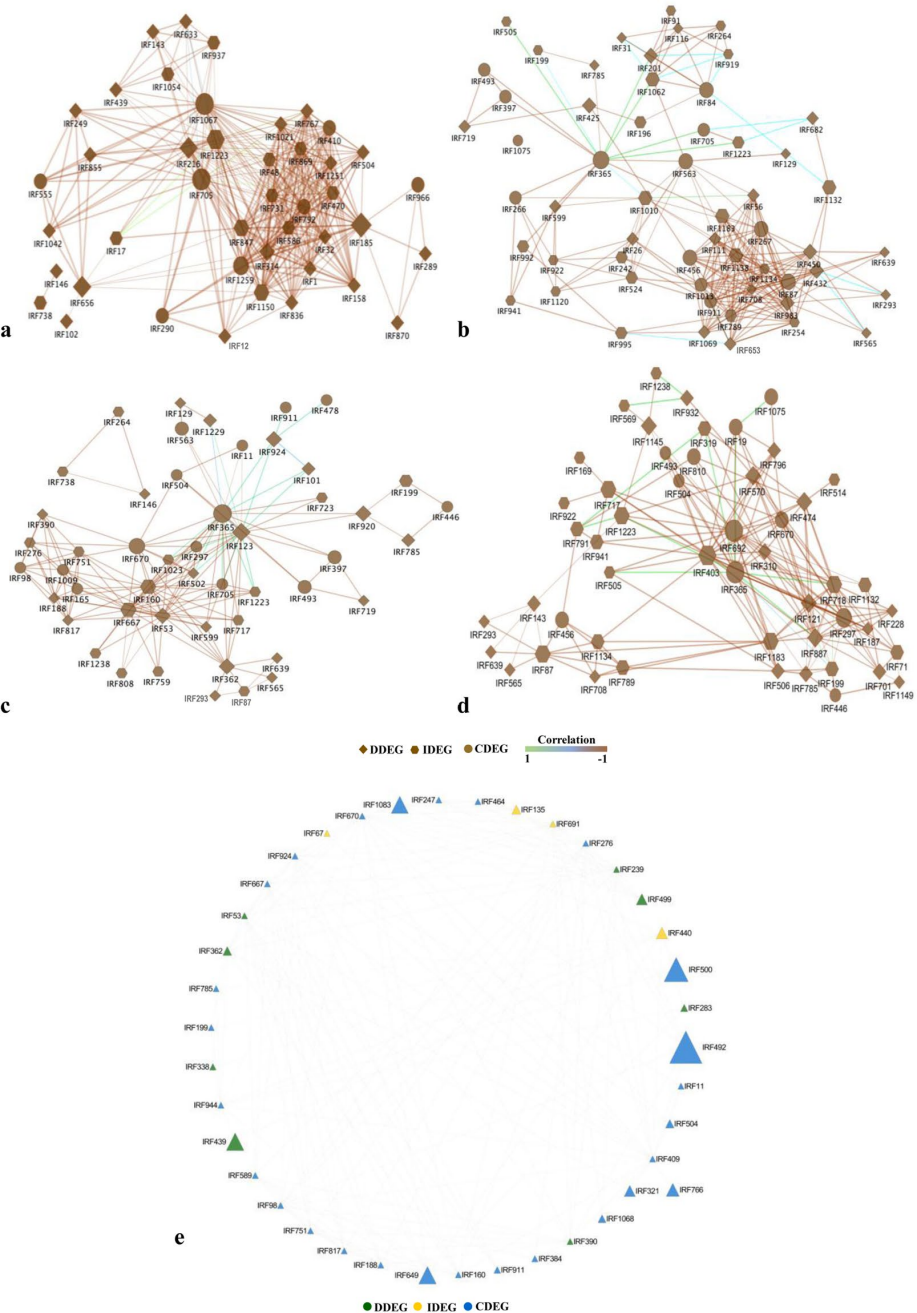
Gene sub-networks associated with pathostress and mapping transcription factor network. (**a**) cell organization and biogenesis, (**b**) transcription, and translation regulation, (**c**) signal transduction, (**d**) nucleic acid processing, cell cycle replication and metabolism response, (**e**) transcription factor network. Nodes and edges represent genes and coexpression between genes, respectively.

Characteristic of disease and immune state can be governed by gene expression dynamics as a cause and a consequence of patho-stress^49^, therefore, we analyzed integrated gene expression profile in two contrasting genotypes to understand functionality and modularity of gene interactions. We found variability in co-regulated gene expression across time points and between genotypes, suggesting that attributes of disease and immunity is dictated by functionality and modularity of gene interactions and often share common and diverse function. Further, we constructed a maximum continuous network consisted of 389 nodes connected via 9079 edges based on temporal gene expression data and known PPIs (Fig. 5). Visual inspection of the resulting interactome revealed highly connected functional and regulatory network and identified key exclusive or specific IRFs that play important role in disease and immune state. Resulting network was densely organized and consisted of four biologically significant coherent subnetworks with functional sub-specialization. These subnetworks were significantly enriched for IRFs belonging to biological pathways involving cell organization and biogenesis (43 nodes, 354 edges) (Post-translational protein modification) (SN1); transcription and translation regulation (SN2) (55 nodes, 235 edges) (Translation); signal transduction (SN3) (50 nodes, 165 edges) (Transcription); Nucleic acid processing, cell cycle replication and metabolism response (SN4) (50 nodes, 180 edges) (Cellular assembly and transport) (Fig. 6a–d; Supplementary Dataset 5).

Subnetwork SN1 was associated with vesicular trafficking and might be involved in sorting of mRNAs which remain untranslated due to downregulation of many ribosomal proteins during stress. Macromolecule biogenesis is known to have prominent role in gene regulation during stress^50^. We found that 20 S proteasome A and B subunit (IRF1042) was upregulated while ubiquitin-conjugating enzyme E2 (IRF12) and 26 S protease regulatory subunit 6 (IRF1054) were downregulated in the JG-62. However, ubiquitin (IRF1204) was first induced and then repressed in both genotypes. Notably, SN1 was also markedly enriched for genes involved in phosphorylation cascade. Many of which are core stress associated signaling component in addition implicated in the regulation of nucleic acid processing, including serine/threonine kinases and other related kinases (IRF1, 48, 185, 410, 470, 586, 656, 792, 1021, 1150, 1251), CIPK (IRF410), leucine-rich repeat (LRR) transmembrane protein kinase (IRF1259), shaggy-related protein kinase gamma (IRF767, 869), calcium-dependent calmodulin-independent kinase (IRF847) and putative receptor-like protein kinase (IRF731). While LRR TM PK (IRF314) is known to play role in plant defense^51^, involvement of APK1 (IRF32) is a novel finding. A few of these kinases were seen to be placed distantly from the central cluster and included protein kinase (PK) domain (IRF870). Both APK1 and PK were induced more in susceptible genotype suggesting that distinct signaling components may be activated in diseased state. Subnetwork SN2 (transcription and translation regulation) linked to SN1 was dominated by ribosomal proteins and translation factors. Ribosomal proteins like L35, L37a, L23, L15, S8 (IRF922, 789, 1134, 505) were downregulated while S13–2 (IRF324) was upregulated in both genotypes. This may be attributed to stress conditions inducing a radical reprogramming to inhibit the translation and promote the repair of defense-related genes. Ubiquitin encoding genes (IRF705, IRF1204) may act as a regulator for activation of many ribosomal proteins which perform distinct roles in specific cellular processes. Ribosomal protein S3a (IRF682) is a key player in cell transformation^52^ while ribosomal protein L27 (IRF254) is involved in mRNA degradation triggered by genotoxic stress^53^. Suppression of the ribosomal L2 (IRF26) was identified as a novel mechanism for stress adaptation^54^. This is consistent with the notion that shared and distinct set point governed by transcription and translation dictates disease or immune response.

Subnetwork SN3 was enriched in GTP binding proteins, chromatin remodelers, ribosomal proteins and ubiquitin factors. Histone variants (histone H3.2) (IRF796) and deacetylases (HDT1, HDA2) (IRF670, IRF504) were clustered along with ubiquitin (IRF1204) and were further connected to GTP binding proteins, namely ADP-ribosylation factor 1 (IRF810) and ribosomal proteins (IRF717, 922, 941, 1075). Our data showed induction of Ras encoding gene (IRF297), golgi SNAP receptor complex member 1 (IRF310) and vesicle sorting (GOS11) (IRF474) during early time points. Two of the histone deacetylase HDT1 (IRF200, 504, 670) showed interaction with uncharacterized protein (IRF1238) that act as versatile modulator of chromatin function and affect the structural flexibility of DNA. Histone deacetylase HDT1 showed downregulation, which indicates that chromatin decondensation drives distinct transcription patterns. Co-regulation and interaction of these genes suggest modulation of signal transduction pathways and plethora of metabolism shared in both genotypes. SN3 was functionally linked to SN4 (nucleic acid processing, cell cycle replication and metabolism response). Association of disease or immunity with cell cycle checkpoint was found for tubulin alpha-1 chain (IRF701) in SN4. It was downregulated in both the genotypes, indicating that cell cycle regulator recycling might play vital role during patho-stress.

Cellular processes are governed by complex gene expression programs regulated by transcription factors (TFs). To identify TF dynamics, we segregated TFs, chromatin-related proteins, transcriptional machinery components and protein kinases and mapped their differential expression into transcriptional regulatory networks (TRNs) (Fig. 6e). Expression based regulatory links predicted by interactions comprised of 39 distinct TFs regulating 186 distinct targets with p < 0.05 supporting that perturbation of TF is directly related to immunity or disease state. We recovered a number of well-characterized complexes, such as zinc finger family, 14–3–3, histones and TF complex. Finally, TF interactions were used to functionally interrogate the mastermind signaling network. Transcription factor reprograming observed in network (39 nodes) contributed centrally to the robustness. Transcription factor bHLH122 (IRF165), zinc finger A20 and AN1 SAP8 (IRF703) and nuclear transcription factor Y subunit A-7 (IRF723) were among the top transcription factor while serine/threonine kinases were among the top protein kinases associated with the DEGs across the datasets. Unique observation was predominant cluster of genes belonged to homeodomain leucine zipper family (IRF667) and BEL1 (IRF160) while the other cluster was centered around bHLH (IRF188, 751, 817, 1048, 1264) alongwith MYB transcription factor MYB2 (IRF203), Hy5 transcription factor (IRF276) and BEL 1 domain (IRF160). Although these TFs shows development related function^55^, their involvement in disease/immunity would open up possibility for exploring their functionality.

### Network specializations of wilt diseaseome and immunome

Analyses of modular network and machine learning methods dissect role of disease/immune-related gene network. We designed an integrative multistep framework combining gene expression analysis and modular co-expression network to systematically characterize the organizing principles of wilt diseasome and immunome.

**Figure 7:**
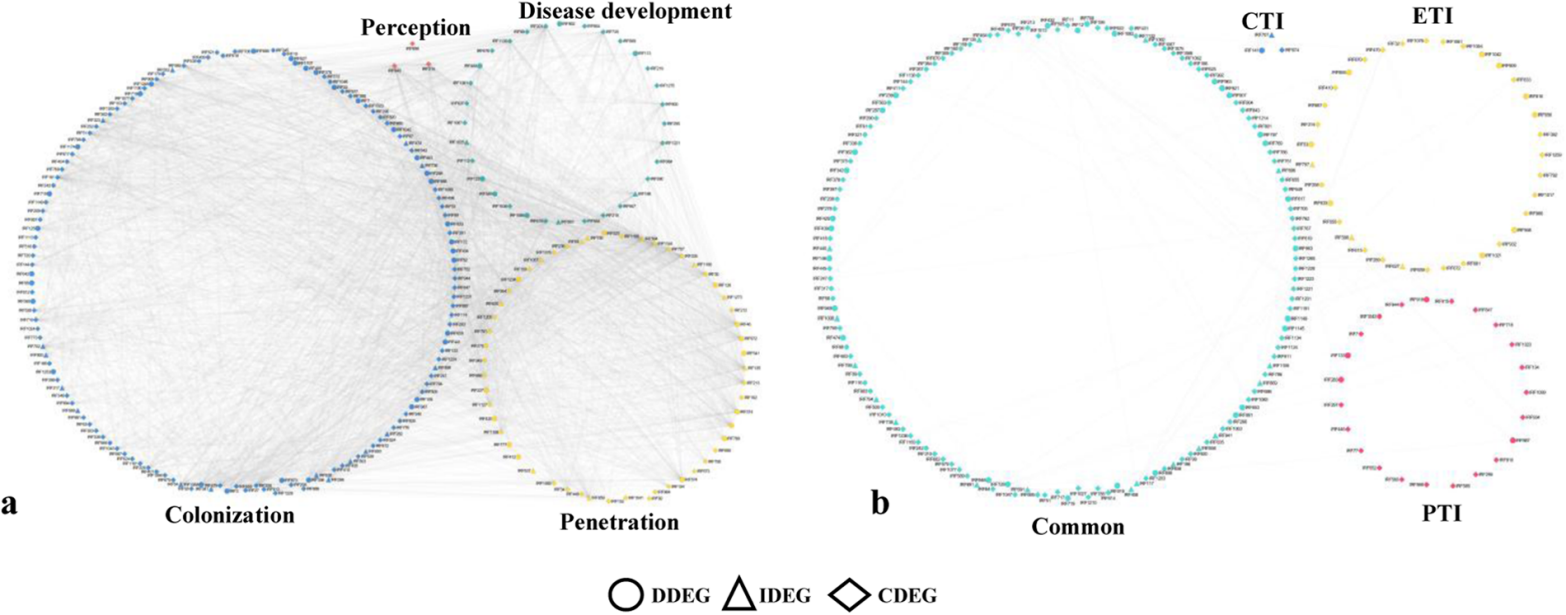
Modular network of wilt diseaseome and immunome. (**a**) wilt diseasome segregated into perception, penetration, colonization and disease development. (**b**) immunome assembled from gene expression data in correlation network framework segregated into four hubs encompassing PTI, ETI and CTI. Nodes and edges denote genes and interactions between genes, respectively.

Each disorder has a definite and discrete genetic origin, and thus the disease network seems to be disconnected into several nodes linked to well-defined events of disease cycle grouped into small hubs of closely related phases^56^. We constructed wilt diseasome having four biologically relevant modules, encompassing pathogen perception, penetration, colonization and disease development (Fig. 7a). Although the wilt diseasome layout was generated based on the knowledge of disease event, major goal was to identify target genes that may function selectively in susceptible genotype. Observed four modules had linear dependencies of 233 statistically significant genes. These modules uncover inconsistencies associated with disease state. Perception related genes (3 DEGs) were associated with module 1 (M1) including receptor-like protein kinase, brassinosteroid insensitive1-associated receptor kinase 1 (BAK1) and LRR receptor-like serine/threonine protein kinase indicative of stress regulation for initiation of disease signaling. Further, heterogeneity and complex association involving calcium-dependent protein kinase 8, nucleoside diphosphate kinase, G-protein signaling elements, phytochrome-associated serine/threonine-protein phosphatase 3 were observed in module 2 (M2) consisting of 54 DEGs for fungal penetration. The most highly active set of colonization associated genes (144 DEGs) like proteases, transferases, ribosome machinery and transporters were abundant in module 3 (M3). Finally, module 4 (M4) was enriched with genes involved in disease development like TFs, coactivators, translocases and signal elements functional in disease pathway. Some of the genes matching these criteria are involved in DNA damage responses and cell cycle checkpoint regulation^57^. Thus, it can be concluded that DEGs associated with signaling components were co-expressed during perception and penetration of *Fusarium*, whereas in colonization protein fate, transport and transcription related DEGs were majorly represented. As expected, varied families of transcription factors and signaling pathways components showed interactions in disease development module. Consistent with morphological and biochemical changes diseasome showed diverse signaling and regulatory pathways in each of the four modules.

Triggering innate immune response reveals homeostatic points that regulate cellular pathway^6^. Although, PTI and ETI are two main plant immune responses to counter pathogen invasion, however, in response to fungal pathogens CTI is considered separately as immune response to chitin polysaccharides of fungal cell wall^9^. To determine immunity-related pathways, we designed and assembled gene expression profiles in correlation network framework delineated into four hubs encompassing PTI, ETI and CTI (Fig. 7b). We adopted a scoring strategy to reduce false positives and remove unreliable gene association data. The network comprised of 207 nodes and 2482 edges was enriched with genes implicated in pattern-, effector- or R-mediated signaling and homeostasis. For PTI, CTI and ETI modules, there were 23, 3 and 35 DEGs, respectively. Also, we identified shared DEGs amongst PTI, CTI and ETI encompassing 146 genes and 916 interactions. The module referred as “common” might represents a core component involved in several biological processes that favour immune response over development.

Immune response and metabolic changes were two major themes in the “common” module. Delineating regulatory interactions between nodes showed major connected components involved in ROS generation, protein fate determination, cell rescue and defense and intruder perception. Minor components in immunome involved in cell wall reinforcement and macromolecular trafficking. Of the three major hubs, hub1 mapped to PAMPs and PRR interaction, including tetratricopeptide repeat protein (IRF7) and putative lipid-transfer protein DIR1 (IRF260); processing and presentation via complex formation encompassing type I inositol 1,4,5-trisphosphate 5-phosphatase CVP2 (IRF163), coronatine-insensitive protein 1 (IRF81) and phospholipase D alpha 1 (IRF544); differentiation, activation and cell receptor signaling comprised of Ras-related protein RABG3f (IRF638), Ras-related protein RABA1b (IRF121), GTP-binding protein SAR1B (IRF319), developmentally-regulated G-protein 3 (IRF1043), calnexin homolog (IRF918) etc. Hub 2 mapped to effector and resistance protein interactions, including receptor kinase mediated signal transducer and transcriptional activator alongwith associated signaling events that include disease resistance response protein DRRG49-C (DRRP; IRF1202), DRRP Pi49 (IRF1186), probable receptor-like protein kinase (IRF1030), BAK1 (IRF495), probable LRR receptor-like serine/threonine-protein kinase (IRF1259). Hub 3 forms molecular signature anchored around extracellular matrix genes associated with chitin mediated activation of immune response comprised of endochitinase (IRF141), beta-glucosidase 13 (IRF1057), probable polygalacturonase (IRF1205) etc. Other expressed genes include fatty acid anchor proteins that links matrix to plasma membrane and transduce the signal to cell interior. By observing PTI modules, we found that metabolism, transcription, signaling and cell rescue and defense related DEGs were the most predominant, whereas in ETI signalling protein fate related DEGs and their association were majorly represented. Interestingly, out of three DEGs in CTI module one of them was endochitinase, a key player in chitin mediated immune response. As proposed by Tsuda and Katagiri (2010), in plants immune-related transcriptional reprogramming is a shared response during PTI and ETI and separate signalling events regulate PTI and ETI.

### Identification of regulatory hubs and subcellular layering in network

**Figure 8:**
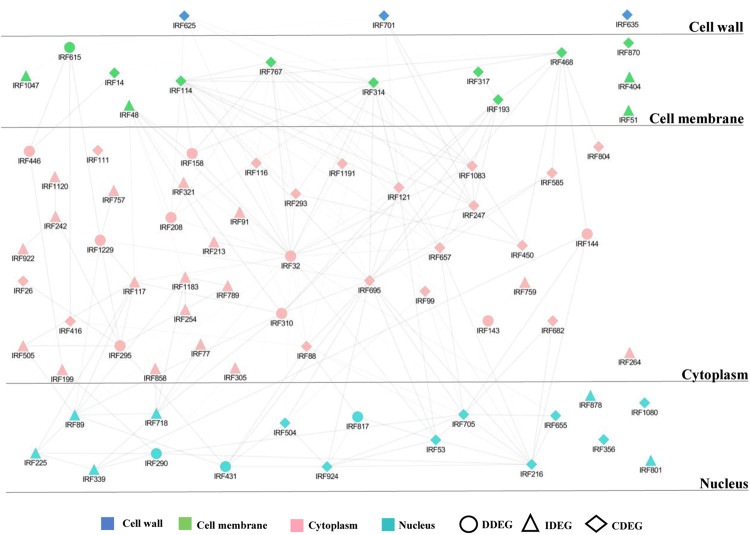
Subcellular layers illustrating the PPI sub-network. Layered PPI network assembled from microarray data was separated into four cellular organelles. Degree of connectivity is mentioned in Supplementary Table S7.

Large number of regulatory genes, their overlapping expression pattern and localization demonstrates complexity of cellular elements to environmental stresses. We built layered interectome to analyze organeller dynamics during fungal invasion. The assembled PPI network from microarray data was divided into four layers: Cell wall (1%), cell membrane (6%), cytoplasm (78%) and nucleus (15%) (Fig. 8; Supplementary Dataset 6). Correlation was estimated for every gene pair using in-house program named COREL_FIND. In order to highlight shared and distinct biological processes governing disease or immunity, we selected gene pairs with a minimum expression value difference of 1.5 for each time point over a minimum of three time points. We pruned the initial network and constructed simpler gene regulatory model with 76 nodes and 197 edges. An important observation was that some hubs were common in both genotypes but showed interaction with different proteins, wherein some of the interacting partners were common.

Next, we evaluated our data to identify the genes co-expressing in at least two time points in either of the genotypes with a difference of 2 between their expression values. The resulting set of genes was analyzed with BINGO to prepare a combined process map (Supplementary Fig. S4). Results showed that translation initiation and elongation seemed to be predominant in susceptible genotype while protein modification, particularly phosphorylation was predominant in the resistant genotype. This suggests that apart from transcriptional control, translational and post translational modifications are major players in mediating plant immune responses.

## Discussion

Interplay of defensive and offensive strategies regulate immune responses in plants against pathogens. Successful invasion suppress basal defense and reprogram host cellular machinery to restructure plant biological processes^9^. Stimulation of chitin, PAMP and effector-triggered immunity (CTI, PTI and ETI) involve cellular reprogramming leading to defense circuits, self-tolerance and pathogen resistance^6,58,59^. Understanding these regulatory processes associated with pathophysiological pathways and host specific resistance remains a challenge but can have important mechanistic implications in crop improvement. In this study, we identify patho-stress responsive genes, molecular pathways and biomarkers relevant for wilt disease and immune response. We performed Go annotation and network-based analysis using Cytoprophet Maximum Likelihood Approach algorithm to generate a probabilistic network model. This analysis assesses the impact of deregulated genes and their interactions on specific biological response. It identifies immune- or disease-responsive genes and highlights advantages of this methodology over available plant systems biology approaches. Compared to other methods of differential gene expression analyses^60^, the major advantage of our strategy is that the gene is included in the network based on prior evidence narrowing the assumption based conclusions of interactions between candidate genes and at the same platform discover novel candidate with known interactions. We identified a large gene co-expression network that was enriched for transcripts related to shared and distinct patho-stress associated processes. In keeping with similar network-based studies of complex diseases of human and yeast, our approach leverages the combined evidences from biochemical analysis and gene expression profiling. Within the network, we identified functionally coherent and coordinated cell organization and transcriptional programmers, signaling hubs, replication, metabolic regulators and modulators segregated into sub-networks. The finding of increased expression of ethylene-responsive TF, zinc finger A20 and AN1 SAP8 and selenium-binding protein 2 in susceptible genotype and its downregulation in resistant genotype is in accordance with earlier reports describing the effect of genotypes in fungal pathogenesis. Therefore, targeting both disease and immune signaling has been proposed as a possible avenue for identification of disease or immune regulators.

Functional feedback loops and hormonal rebalancing is achieved through collective activities of genes and its product^61^. System level analysis of biological processes provides an endeavor to assess interactions between molecular entities^60^. Pathways significantly enriched in CDEGs, IDEGs and DDEGs include family of 40 S ribosomal proteins, 60 S ribosomal proteins and ubiquitin suggesting that shared functional features provide insight into the fundamental aspects of cell organization during patho-stress. Surprisingly, GO analysis performed on active network showed significant representation of certain transcripts, namely agglutinin (IRF884), allantoinase (IRF1072) and neutral ceramidase (IRF179), which showed variations in fold-change, form, or cellular compartmentalization. These non-canonical proteins might be a new potential target for biomarker discovery. We also found many shared and distinct protein kinases in the co-regulatory network those might be involved in perceiving and relaying the signals to the downstream components. In our data, protein kinase (IRF114) was connected with ribosomal protein S10 (IRF116), T-complex protein 1β (IRF757), elongation factor (IRF1229) and GTP binding protein indicating that this may regulate protein synthesis in stress. Also protein kinase (IRF114) showed interaction with cell division protease (IRF143) through translation initiation factor 5 A (IRF293) that might have role in modeling cell division during stress. Yet another hub protein kinase, namely shaggy-related protein kinase gamma (IRF767) interacted with polyadenylate-binding protein (IRF695) and MyB transcription factor (IRF924). RNA binding protein and Myb further interact with ubiquitin (IRF705) and HDA2 (IRF504). Cross interactions suggest that shaggy-related protein kinase gamma might regulate defense response at transcriptional and post transcriptional level. We also identified shared coregulated hubs in both the genotypes enriched in APK protein kinase (IRF32), kinetochore SKP1 (IRF290), elongation factor EF1 (IRF1229) and DnaJ (IRF615). APK1 occupied the central position and was connected to oligosaccharyl transferase (IRF158) and peptidyl-prolyl cis-trans isomerase (IRF99) through putative leucine repeat protein (IRF657) suggesting its role in protein modifications. Further, APK1 was connected to kinetochore SKP1 (IRF290) through ubiquitin (IRF705) and DnaJ (IRF615) through elongation factor 1 A protein (IRF1229). Association of these proteins might be required to impart resistance to plants. Interestingly, APK1 was found to be connected to subtilisin-like protease (IRF625) and inorganic phosphate transporter PHO84 (IRF468) of fungal origin, suggesting thereby pathogen induced phosphorus starvation mediated by APK1 in plant during fungal stress. We also found syntaxin (IRF193) highly connected to cystatin (IRF208) through APK1 Kinase (IRF32). Interaction between syntaxin and cystatin suggests that syntaxin might be involved in secretion of cystatin, an antifungal protein to the cell periphery during defense response. Regulatory hubs coregulated only in the resistant genotype included serine/threonine protein kinase (IRF48), glutathione S transferase (IRF117), Ras GTPase (IRF718) and putative DNA binding protein (IRF863). Aquaporin (IRF2132) had connections with unknown protein (IRF505) alongwith GTP binding protein, namely obg ATPase (IRF295) and putative DNA binding protein (IRF89). This DNA binding protein was also connected to ribosomal proteins (IRF505, 1183) and a Ras GTPase (IRF718). Interconnection of putative DNA binding protein (IRF89) with ribosomal proteins (IRF505, 1183) and Ras GTPase (IRF718) reflected that translation processes were governed by signal transduction and protein-DNA interactions during stress. Results showed that translation initiation and elongation seemed to be predominant in susceptible genotype while protein modification, particularly phosphorylation was predominant in the resistant genotype. This suggests that apart from transcriptional control, translational and post translational modifications play a significant role in plant immunity. Dissection of shared and distinct aspects of immunity and susceptibility is valuable and can provide important insight for breeding disease-resistant crops. Further, defining the nature of causative and descriptive markers is a major challenge. To investigate causative and descriptive markers for immunity or disease response, we focused on immune adaptation and disease development events, including hormone and signaling response, accumulation and allocation of biomolecules and effect on cellular organization. Subtle expression changes occur from perception until colonization and disease state in JG-62, whereas WR-315 exhibit a modular PTI, ETI or CTI immunity. In the immune state, more prominent genes were peroxidase, PAL, NAD(P)H-dependent 6′-deoxychalcone synthase, PR10 can be observed as descriptive markers. Transcription factor bHLH115, heat shock transcription factor B2A, probable methyltransferase PMT21, NOT2/NOT3/NOT5 and AT-rich interactive domain-containing protein 3 are uniquely characterized by a coordinated regulation in both genotypes and might have implications in disease development and thus can be considered as causative markers. Instead, genes exhibiting up/downregulation in both genotypes like APK1A, Zinc finger protein (GATA-type) BEL1-like homeodomain transcription factor and probable nucleolar protein 5–2 are associated with cellular reprogramming and can be causative or descriptive markers. Genes allied with disease progression (e.g. NAD(P)H-dependent 6′-deoxychalcone synthase, PMT21, NOT2/NOT3/NOT5) and immunity manifestation (e.g. PAL, peroxidase, PR10) both spatially and temporally might be associated with biomarker development.

Taken together, our data provide the first evidence of transcriptional plasticity in regulating disease and immune pathways using gene expression changes and network analysis in wilt disease. We identified disease or immune pathways previously reported to be relevant, as well as novel potential players of *Fusarium* pathogenesis. Strikingly, we explored a balanced homeostatic innate immune and disease response and demonstrated changes in signal signature and sharing of primary metabolic components in defense and disease. More generally, the network framework described here can be employed across diverse diseases and host range.

## Methods

### Plant material and *in planta* infection

Chickpea (*Cicer arietinum*. *L*) seeds of wilt susceptible genotype JG-62 and resistant genotype WR-315 were surface sterilized. Seedlings were grown at 25 ± 2 °C on MS basal medium (agar 0.6% w/w) under 16 h/8 h (light/dark) photoperiod with 50 ± 5% relative humidity as described by Ashraf *et al*.^31^. *Fusarium oxysporum ciceri Race 1* (*Foc1*) was cultured at 28 °C in potato dextrose broth. After 3 weeks of germination, seedlings were treated with either *Fusarium* spore suspension (1 × 10^6^/ml) or water as control. Both control and infected plants from both the genotypes were grown under same conditions. Root and collar tissues were collected at 6, 12, 24, 48 h and 5 days post inoculation. All the samples were snap-freezed in liquid nitrogen before storing at −80 °C. Experiments were performed thrice for three independent biological replicates.

### Physiological and biochemical analyses

Physiological and biochemical analyses were performed for control and fungal treated chickpea root tissue collected after post-infection time points for both genotypes. RWC was measured by determining dry weight (DW), fresh weight (DW) and turgid weight (TW) of each sample as described in (20). Ions leaching into the MQ water were measured to estimate electrolyte leakage from the leaf sections. Samples placed in MQ were incubated for 4 h at room temperature and conductivity before (C1) and after (C2) autoclaving was recorded using conductivity^62^. Experiments were carried out in triplicates. Free proline content, photosynthetic pigments and lipid peroxidation were measured against corrected weight of the actual moisture content of tissue as described earlier^62^. All the experiments were repeated three times.

### DAB Staining

ROS was detected using 3,3′ diaminobenzidine (DAB) staining. In brief, chickpea seedlings of resistant and susceptible genotypes were treated with *F*. *oxysproum* and the roots were stained in 1 mg/mL of DAB buffer solution and kept for 10 h at RT. After staining, the tissues were boiled in acetic acid and lactophenol for 2 min and rinsed with 50% ethanol for 5 times. Finally, the roots were observed under Nikon Eclipse 80i Microscope (Nikon).

### Quantification of *F*. *oxysporum* DNA

Genomic DNA was extracted from infected plant roots using DNeasy Plant Mini Kit (Qiagen). Real-time PCR was performed with *Fusarium* specific as well as chickpea specific gene primers and the normalized Ct values were used for the estimation of fungal biomass. The amplification and quantification of *F*. *oxysporum* DNA was conducted using primers spanning internal transcribed sequences of fungal gene. Approximately, 20 ng of genomic DNA was used as the template for each sample. Sequence of the primers used in the study is given in Supplementary Table S1.

Microarray analysis. Amplification of cDNA clones from the susceptible and resistant subtracted cDNA libraries and preparation of cDNA microarrays has been done from two biological replicates as described previously by Ashraf *et al*.^31^. cDNA microarray having 6072 probes that correspond to 1749 unigenes, including 166 unigenes which showed homology to *Fusarium* sp. RNA from control and *Fusarium* infected WR-315 and JG-62 roots at different post infection time points was extracted using Trizol reagent (Invitrogen, CA) and reverse-transcribed followed by labelling of cDNA samples with Cy3 and Cy5 fluorescent dyes, respectively. Hybridization of purified cDNA onto microarray slides were carried out in hybridization chambers (Corning, USA) at 65 °C for 16 h as described in Ashraf *et al*.^31^. Scan array 5000 scanner and Scan array express software (PerkinElmer, MA) was used for scanning and analyzing microarrays. Raw data was processed by excluding spots of poor morphology, high local background, bubbles and channel intensities less than 500 for further analysis. Background correction by subtracting local background intensity of each spot from its foreground intensity value and intensity dependent Lowess normalization was performed using Avadis software (PerkinElmer, MA). Quantification of spots were performed by an adaptive method and Cy5/Cy3 signal ratio was obtained as described previously^31^. Significance of the data was calculated by Benjamini and Horchberg FDR for multiple corrections in cross-slide one class t-test on two biological replicates hybridized on two microarray slides each having individual clones spotted in duplicates with p ≤ 0.05 and fold change of 2.5.

### Statistical analysis

We used MEV software (TIGR) for K means clustering analysis of 1287 genes showing altered expression in at least one time point in one of the genotypes. Expression values of the individual gene measured in two genotypes represented as independent sets of values were used for analysis to identify the similarity and differences in expression pattern between two contrasting chickpea genotypes. Principal component analysis and two-way ANOVA was performed on the same set of genes using the same program. The PCA was done with the parameters SAMPLE SELECTION as Cluster genes and CENTERING MODE as Mean.

### Construction of regulatory gene networks

We have built network based on the microarray analysis in association with known protein-protein interaction data from (Database of Interacting Proteins) DIP databases in absence of chickpea interactome. We followed a following strategy: homology search of EST sequences associated with the microarray probe IDs was performed using BLASTx against the SwissProt database. The top SwissProt BLAST hit was in turn assigned to each EST sequence and consequently to the corressponding probe IDs. GO ontology mapping and annotation was also performed for the sequences using BLAST2GO software^46^. A table of SWISS PROT IDs and the corresponding expression data was submitted to Cytoprophet Network inference plugin of Cytoscape. The Cytoprophet Maximum Likelihood Approach algorithm^63^ was used to generate a probabilistic model of network on 572 differential genes falling under regulatory category where the network assigned 391 proteins within it rejecting others on the basis of lack of interaction data or low probability score on which further emphasis was given. Further we submitted the assigned SwissProt IDs assigned to the cytoprophet plugin in Cytoscape (https://www.ncbi.nlm.nih.gov/pubmed/18653520) which computes the probability of two proteins to potentially interact with each other with DIP (https://www.ncbi.nlm.nih.gov/pubmed/11752321) as its reference. Further, we have built diseasome and immmunome based on the microarray analysis. Modules were computed on the PPI network using the cytoscape MCODE plugin (https://www.ncbi.nlm.nih.gov/pmc/articles/PMC149346/), which clusters nodes in a network based on several parameters of their connectivity with all other nodes. GO ontology mapping and literature search was performed on susceptible and resistant genotype datasets. SWISS PROT IDs and the corresponding expression data was submitted to Cytoscape and analysed using the Network Analyser plug in. Further, Network Analyser plug in was used to designate network genes in the respective modules. Plant transcription factor database (Plant TFDB) was used to perform regulatory gene network analysis to identify regulators responsible for observed patterns in gene expression. A culmination of the Cytoprophet and BLAST2GO results was brought about through BINGO plugin of Cytoscape^64^. The complex network of 389 EST’s was supplied to BINGO along with BLAST2GO mapping data in form of a BINGO annotation file. From the BINGO run we got the results in form of statistically prominent biological process groups as per GO Ontology within the network predicted. These groups were divided into four major categories each including a sub category best suiting its parent.

### Construction of subcellular layered network

Apart from the network groups, the complete microarray expression data for all EST’s falling under regulation class were analyzed with a simple self-developed program called COREL_FIND. For a set of microarray expression values it gives an output of probable co-expressing pair of candidates if provided a threshold difference to be considered between the expression values for two genes at same time point and a minimum number of times such threshold is crossed across a time series. This program was used on five time point expression values of susceptible and resistant genotype with a parameter of Difference = 1.5 and Minimum Number of Hits = 3. The set of results obtained were transformed into a single column of non-redundant ID’s and superimposed on the topology of the network previously generated. For these ID’s Statistical Betweeness score was calculated by CentiScale Cytoscape plugin within the main regulation network. The sub-network for these ID’s was extracted from the main regulation network. The nodes with a high betweeness score were predicted to be playing an active role in the plant immunity system.

This program was again used on five time point expression values of susceptible and resistant genotype with a parameter of Difference = 2 and Minimum Number of Hits = 2 to identify the genes co-expressing in at least two time points in either of the genotypes with a difference of 2 between their expression values. The resulting set of genes was analyzed with BINGO to prepare a combined process map.

### Network visualization

Networks were visualized in cytoscape and its plugins. However, to create shared network, output of GO enrichment obtained from BiNGO was imported into Enrichment Map plugin and was visualized in cerebral plugins (http://www.pathogenomics.ca/cerebral/) in pathway style.

### Real time PCR

Expression profiles of few genes selected from the microarray dataset were validated by performing quantitative real time PCR. For each genotype and time point, total RNA quantification was done on a NanoDrop Spectrophotometer (Nanodrop Technologies) and 5 µg of total RNA was used for cDNA preparation using Reverse transcriptase kit (Applied biosystems). For qRT-PCR, cDNA was diluted ten times and reaction mixture was prepared for individual genes in triplicates using Sybr Green Mastermix (Applied biosystems). PCR was performed on an ABI 7500 sequence detection system according to the manufacturer’s protocol (Applied Biosystems). ^ΔΔ^Ct method was used for relative quantification. Normalization of the data was done using 18 S as an endogenous control. Primers sequences are listed in Supplementary Table S1.

### Data availability statement

All data generated or analysed during this study are included in this published article (and its Supplementary Information files).

## Electronic supplementary material

Supplementary Information

Supplementary Dataset 1

Supplementary Dataset 2

Supplementary Dataset 3

Supplementary Dataset 4

Supplementary Dataset 5

Supplementary Dataset 6
