# Supplementary material for: Integrative network analyses of wilt transcriptome in chickpea reveal genotype dependent regulatory hubs in immunity and susceptibility
Source: Sci Rep. 2018 Apr 25;8:6528. doi: 10.1038/s41598-018-19919-5 (PMC5916944; doi:10.1038/s41598-018-19919-5)
Supplement: Supplementary file 1 — Supplementary Information [file 41598_2018_19919_MOESM1_ESM.pdf]

Title

Integrative network analyses of wilt transcriptome in chickpea reveal genotype dependent regulatory hubs in immunity and susceptibility

Authors:

Nasheeman Ashraf, Swaraj Basu, Kanika Narula, Sudip Ghosh, Rajul Tayal, Nagaraju Gangisetty, Sushmita Biswas, Pooja R. Aggarwal, Niranjan Chakraborty and Subhra Chakraborty*

Affiliation: National Institute of Plant Genome Research, Aruna Asaf Ali Marg, New Delhi-110067

*Corresponding Author:
Dr. Subhra Chakraborty
National Institute of Plant Genome Research
Aruna Asaf Ali Marg
New Delhi, India
Phone: +91-11-26735186
Fax: +91-11-26741658
Email: subhrac@hotmail.com

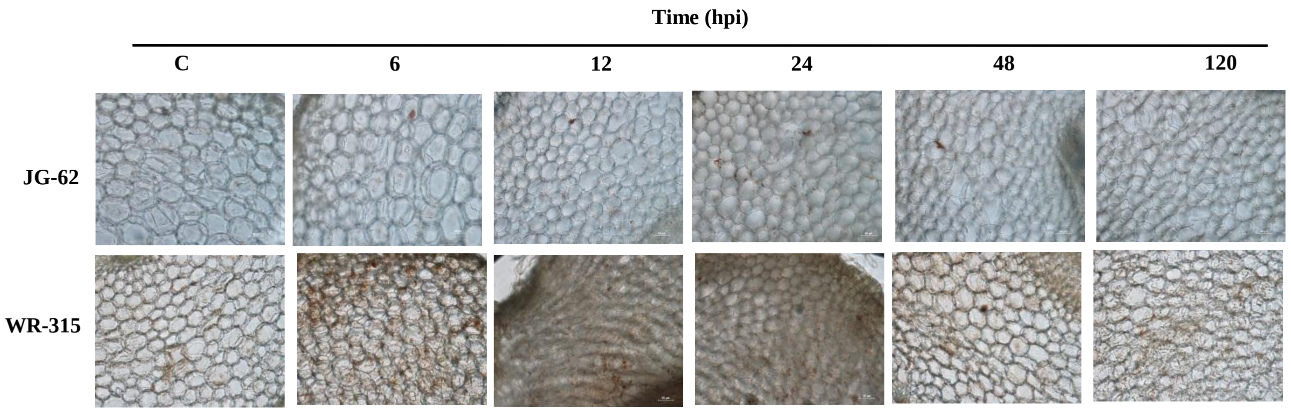

Supplementary Figure S1. ROS production and progression of *F. oxysporum* in the roots of JG-62 and WR-315 plants. *F. oxysporum*-infected roots of JG-62 and WR-315 stained with DAB.

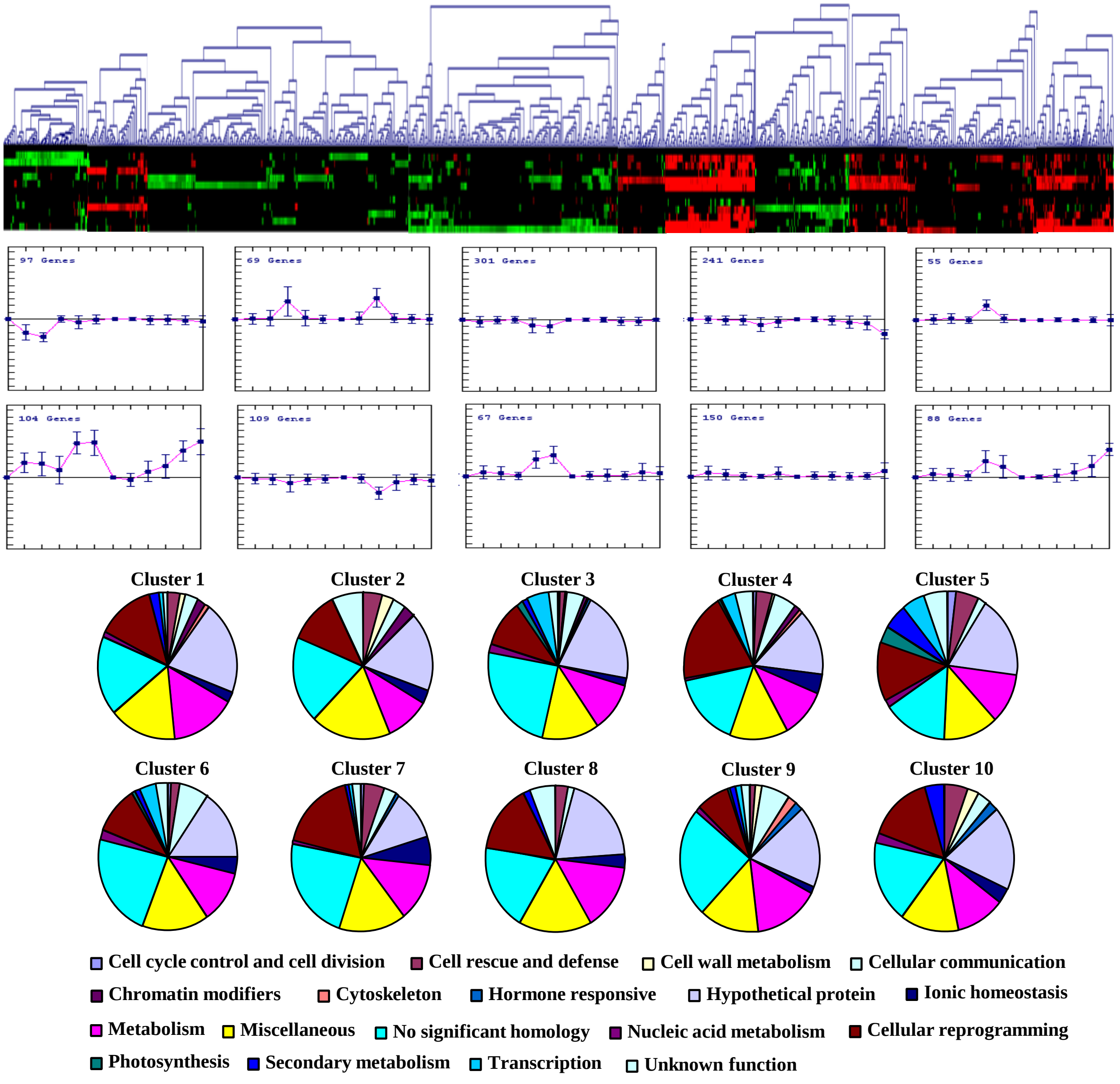

Supplementary Figure S2. Clusterogram of DEGs. DEGs were grouped into 10 clusters based on their expression pattern. The SOTA cluster tree is shown at the top, and the expression pattern in clusters are shown below. High (or low) abundance ranges from pale to saturated red (or green).

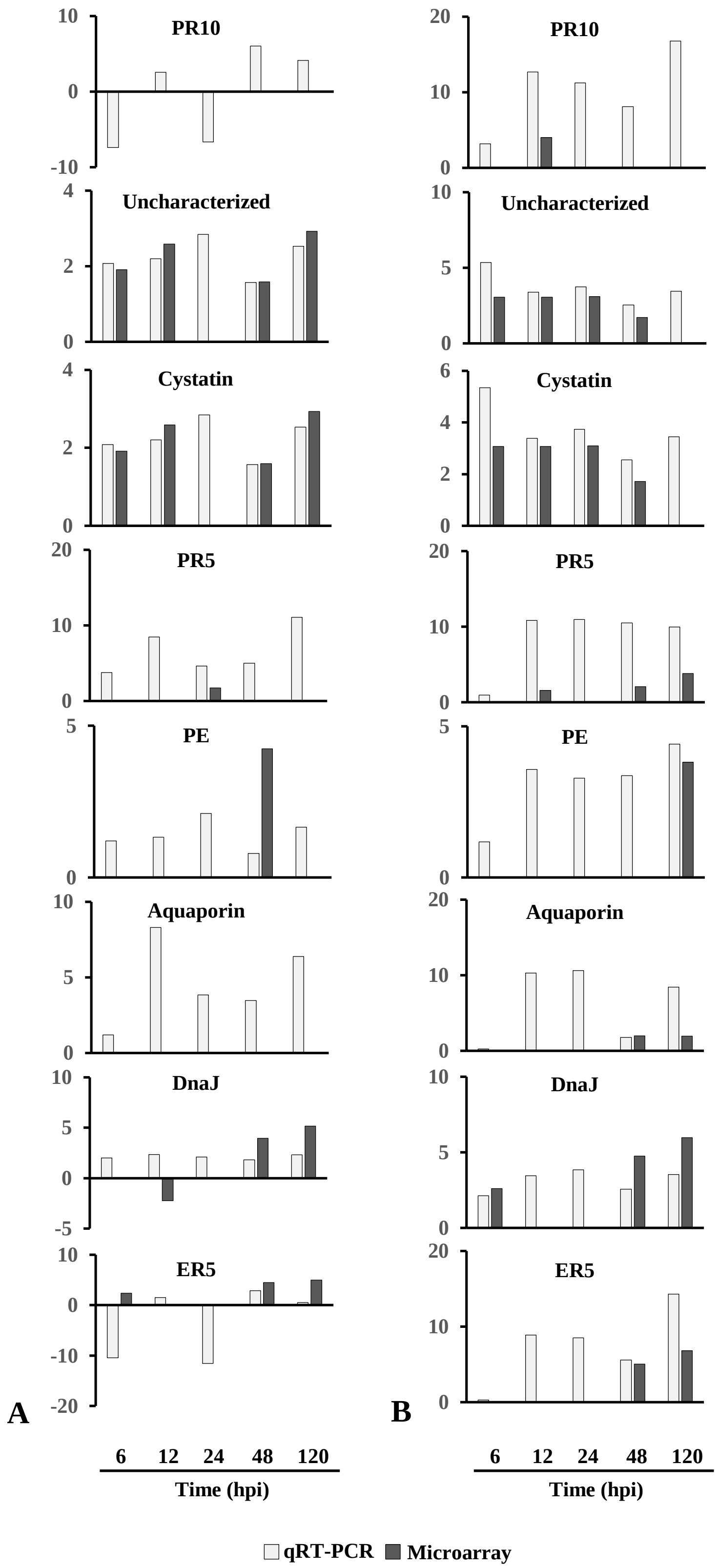

Supplementary Figure S3. Co-relation of gene expression levels by qRT-PCR and microarray experiments. (A) JG-62 and (B) WR-315. Log_2 qRT-PCR expression were compared with the differential expression from Log_2 microarray data.

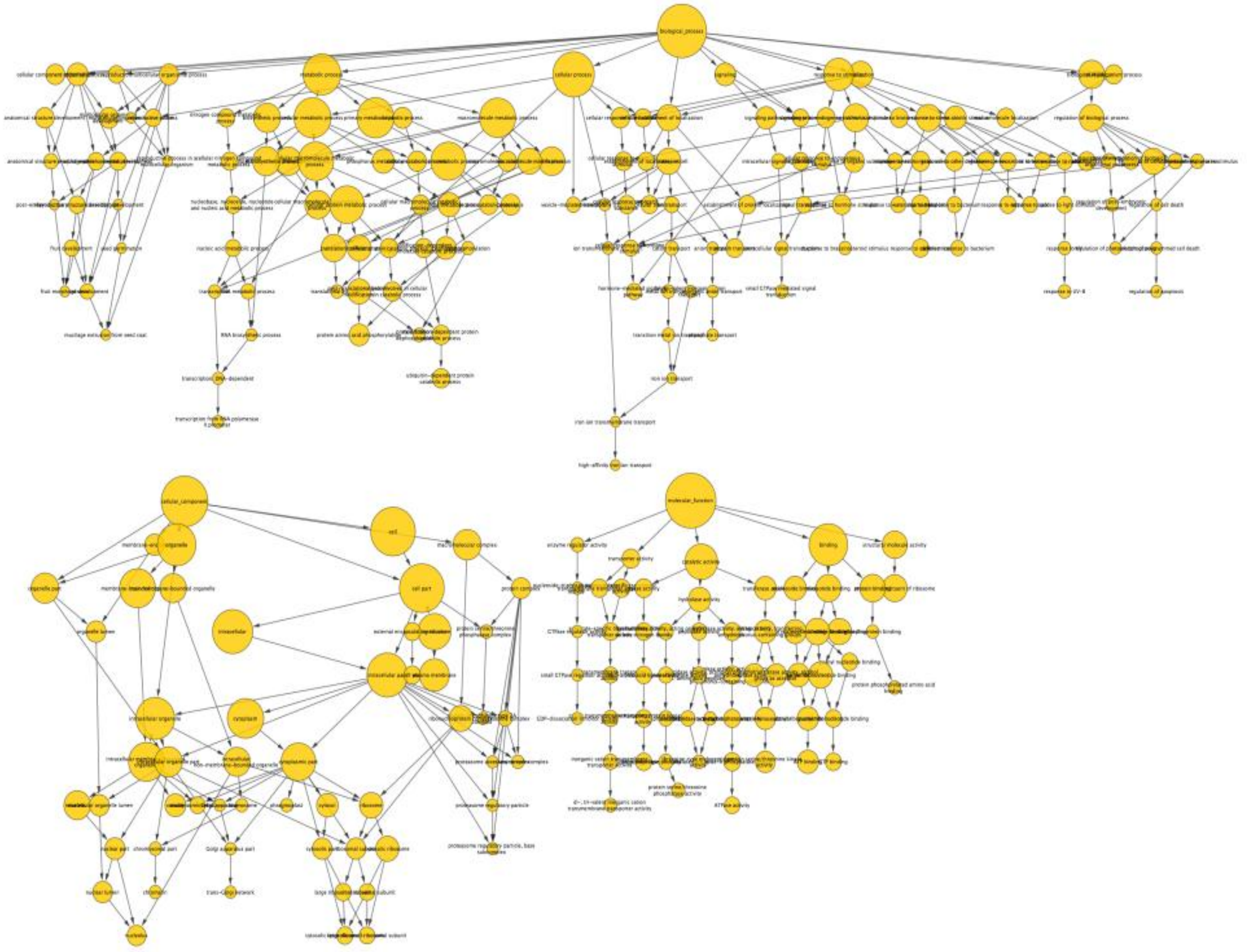

Supplementary Figure S4. Function and coexpression analysis. BLAST2GO results were merged through BINGO plugin of Cytoscape. The complex network of 389 EST's was subjected to BINGO along with BLAST2GO mapping data in form of a BINGO annotation file. These groups were divided into four major categories each including a sub category best suiting its parent.

Supplementary Table S1. Sequence of oligonucleotide primers used for quantitative real time PCR.

Name of primer	Sequence of primer
PR10 - F	5’-TCCTTCACCATCCAGCATACC-3’
PR10 - R	5’-TGACGGGCTCGAGAAAATACA-3’
ERF - F	5’-GGGCAGGTGTCGACTCTCAT-3’
ERF - R	5’-CGAGTCATGGTTGTTGATGGA-3’
Aquaporin - F	5’-TCATCGGCAGTGTTGGAGACT-3’
Aquaporin - R	5’-AACAGCGCTTTAAATCAGGAGAA-3’
PR5 - F	5’-CCGTGTTAGGAGCGGGATTT-3’
PR5 - R	5’-CATCCCCGCAGTTTTGTGT-3’
DnaJ - F	5’-GCTTCCAGTTTACTGTCACACATCTT-3’
DnaJ - R	5’-TTCCCCAGGGTTTGATTTGA-3’
Cys - F	5’-CTTGGTGGAGTTCGCGATGT-3’
Cys - R	5’-TAGCGGGCGAGACTATCGAT-3’
Uncharaterized- F	5’-CGGATGCATTTGAGAAAAACC-3’
Uncharaterized - R	5’-GGAAGAGCCTTATCCAGTGAAATC-3’
PE - F	5’-GCAGATTGCTCATCCCACAA-3’
PE - R	5’-TGTCTAGCATGATGGAGCAGTTTT-3’
FoGPD*_F	5’-AAGGGTGCTTCTTACGACCA-3’
FoGPD*_R	5’-ATCGGAGGAGACAACATCGT-3’
Ca18s_F#	5’-CTCGGCCCAACTCCGGTTCG-3’
Ca18s_R#	5’-CGCACGAAAACCGTCTCCGGT-3’

* FoGPD represents *Fusarium oxysporum* glyceraldehyde3-phosphate dehydrogenase.
# 18S was used as an endogenous control.

Supplementary Reference

1. Assmann, S. M. Heterotrimeric and unconventional GTP binding proteins in plant cell signaling. *Plant Cell* **14,** 355 (2002).
2. Baidyaroy, D., Brosch, G., Graessle, S., Trojer, P. & Walton, J. D. Characterization of inhibitor-resistant histone deacetylase activity in plant-pathogenic fungi. *Eukaryot. Cell* **1,** 538–547 (2002).
3. Bhattacharyya, S. N., Habermacher, R., Martine, U., Closs, E. I. & Filipowicz, W. Relief of microRNA-Mediated Translational Repression in Human Cells Subjected to Stress. *Cell.* **125**, 1111–1124 (2006).
4. Bridges, D. & Moorhead, G. B. G. 14-3-3 Proteins: a Number of Functions for a Numbered Protein. *Sci. STKE* **2005,** re10 (2005).
5. Carvalho, C. M. *et al.* Regulated nuclear trafficking of rpL10A mediated by NIK1 represents a defense strategy of plant cells against virus. *PLoS Pathog.* **4,** (2008).
6. De La Torre, F. *et al.* The tomato calcium sensor Cbl10 and its interacting protein kinase Cipk6 define a signaling pathway in plant immunity. *Plant Cell* **25,** 2748–2764 (2013).
7. Feng, B. *et al.* Protein Poly(ADP-ribosyl)ation Regulates Arabidopsis Immune Gene Expression and Defense Responses. *PLoS Genet.* **11,** (2015).
8. Fu, H., Doelling, J. H., Arendt, C. S., Hochstrasser, M. & Vierstra, R. D. Molecular organization of the 20S proteasome gene family from Arabidopsis thaliana. *Genetics* 149, 677–692 (1998).
9. Gupta, S. K., Rai, A. K., Kanwar, S. S. & Sharma, T. R. Comparative analysis of zinc finger proteins involved in plant disease resistance. *PLoS One* **7,** (2012).
10. Hardie, D. G. Plant protein serine threonine kinases: classification and functions. *Annu. Rev. Plant Physiol. Plant Mol. Biol.* **50,** 97–131 (1999).
11. Kennedy, I. M., Burdon, R. H. & Leader, D. P. Heat shock causes diverse changes in the phosphorylation of the ribosomal proteins of mammalian cells. *FEBS Lett.* **169,** 267–273 (1984).
12. Khan, M. S. & Hemalatha, S. Autophagy : Molecular Insight and Role in Plant Programmed Cell Death and Defense Mechanism. *Int. Res. J. Biol. Sci.* **4,** 78–83 (2015).
13. Naora, H. & Naora, H. Involvement of ribosomal proteins in regulating cell growth and apoptosis: Translational modulation or recruitment for extraribosomal activity? *Immunol. Cell Biol.* 77**,** 197–205 (1999).
14. Pajerowska-Mukhtar, K. & Dong, X. A kiss of death--proteasome-mediated membrane fusion and programmed cell death in plant defense against bacterial infection. *Genes Dev.* **23,** 2449–2454 (2009).
15. Park, M. H. The post-translational synthesis of a polyamine-derived amino acid, hypusine, in the eukaryotic translation initiation factor 5A (eIF5A). *J. Biochem.* **139**, 161–169 (2006).
16. Romeis, T., Ludwig, A. A., Martin, R. & Jones, J. D. Calcium-dependent protein kinases play an essential role in a plant defence response. *EMBO J.* **20,** 5556–5567 (2001).
17. Vashisht, A. A. & Tuteja, N. Stress responsive DEAD-box helicases: A new pathway to engineer plant stress tolerance. *J. Photochem. Photobiol. B Biol.* **84,** 150–160 (2006).

18. Xu, S.-L., Rahman, A., Baskin, T. I. & Kieber, J. J. Two Leucine-Rich Repeat Receptor Kinases Mediate Signaling, Linking Cell Wall Biosynthesis and ACC Synthase in Arabidopsis. *Plant Cell*. **20**, 3065–3079 (2008).
19. Yang, C., Zhang, C., Dittman, J. D. & Whitham, S. A. Differential requirement of ribosomal protein S6 by plant RNA viruses with different translation initiation strategies. *Virology* **390,** 163–173 (2009).
20. Yang, Z., Small GTPases: Versatile Signaling Switches in Plants. *Plant Cell* ***14***, S375–S388, (2002).
